# History of scrub typhus in Indonesia

**DOI:** 10.1093/trstmh/traf017

**Published:** 2025-02-26

**Authors:** Kartika Saraswati, J Kevin Baird, Stuart D Blacksell, Marlous L Grijsen, Nicholas P J Day

**Affiliations:** Oxford University Clinical Research Unit Indonesia, Faculty of Medicine, Universitas Indonesia, 10430 Jakarta, Indonesia; Mahidol Oxford Tropical Medicine Research Unit, Faculty of Tropical Medicine, Mahidol University, 10400 Bangkok, Thailand; Centre for Tropical Medicine and Global Health, Nuffield Department of Medicine, University of Oxford, OX3 7LG Oxford, UK; Saw Swee Hock School of Public Health, National University of Singapore, 117549 Singapore; Oxford University Clinical Research Unit Indonesia, Faculty of Medicine, Universitas Indonesia, 10430 Jakarta, Indonesia; Centre for Tropical Medicine and Global Health, Nuffield Department of Medicine, University of Oxford, OX3 7LG Oxford, UK; Mahidol Oxford Tropical Medicine Research Unit, Faculty of Tropical Medicine, Mahidol University, 10400 Bangkok, Thailand; Centre for Tropical Medicine and Global Health, Nuffield Department of Medicine, University of Oxford, OX3 7LG Oxford, UK; Oxford University Clinical Research Unit Indonesia, Faculty of Medicine, Universitas Indonesia, 10430 Jakarta, Indonesia; Centre for Tropical Medicine and Global Health, Nuffield Department of Medicine, University of Oxford, OX3 7LG Oxford, UK; Mahidol Oxford Tropical Medicine Research Unit, Faculty of Tropical Medicine, Mahidol University, 10400 Bangkok, Thailand; Centre for Tropical Medicine and Global Health, Nuffield Department of Medicine, University of Oxford, OX3 7LG Oxford, UK

**Keywords:** history, Indonesia, *Orientia*, scrub typhus, tsutsugamushi

## Abstract

Scrub typhus is a common but underrecognized cause of fever in the Asia-Pacific region. This review is the first to examine the history of scrub typhus in the context of notable historical events in Indonesia. Scrub typhus was first observed in 1902 and has since been documented through colonial and modern times. However, the available evidence is sparse. This lack of data is influenced by wider factors, including geopolitical climate and socio-economic factors. During the colonial era and World War II, research focused on economic and military interests. There were research gaps during the unstable period following independence in 1945. More research commenced only in the 1970s, mainly under the auspices of the Ministry of Health. Since 2000, there have been sporadic attempts to study scrub typhus on several major islands (Java, Sumatra, Sulawesi, Borneo, Bali). We found 51 relevant articles documenting the presence of the pathogen and its vectors, with only a single case confirmed with standard laboratory testing. This lack of data, combined with low awareness and diagnostic capacity, makes it difficult for policymakers to appreciate the impact of scrub typhus. Indonesia needs sustainable and continuous surveillance systems, infrastructure and research funding to ensure diseases of public health importance are not neglected.

## Introduction

Scrub typhus is an underrecognized cause of acute fever. It is a mite-borne infection caused by intracellular rickettsial bacteria of the genus *Orientia*. It mainly presents as an acute undifferentiated febrile illness, making clinical diagnosis difficult and accurate and affordable laboratory tests are not widely available. The aim of this review is to describe the history of scrub typhus in Indonesia. Through this, we hope to improve the understanding of scrub typhus in Indonesia, suggest areas for further research and inform public health policies that can effectively address the challenges scrub typhus poses in Indonesia. This article focuses on the history and how other factors, such as geopolitical climate and socio-economic status, have affected scrub typhus research in Indonesia. We searched electronic databases, websites and libraries to identify relevant articles using search strategies as previously described.^1^ We included 51 articles detailing the presence of the scrub typhus pathogen and vectors to summarise the history of scrub typhus in Indonesia.

## Late colonial era (up to 1941)

**Figure 1.**
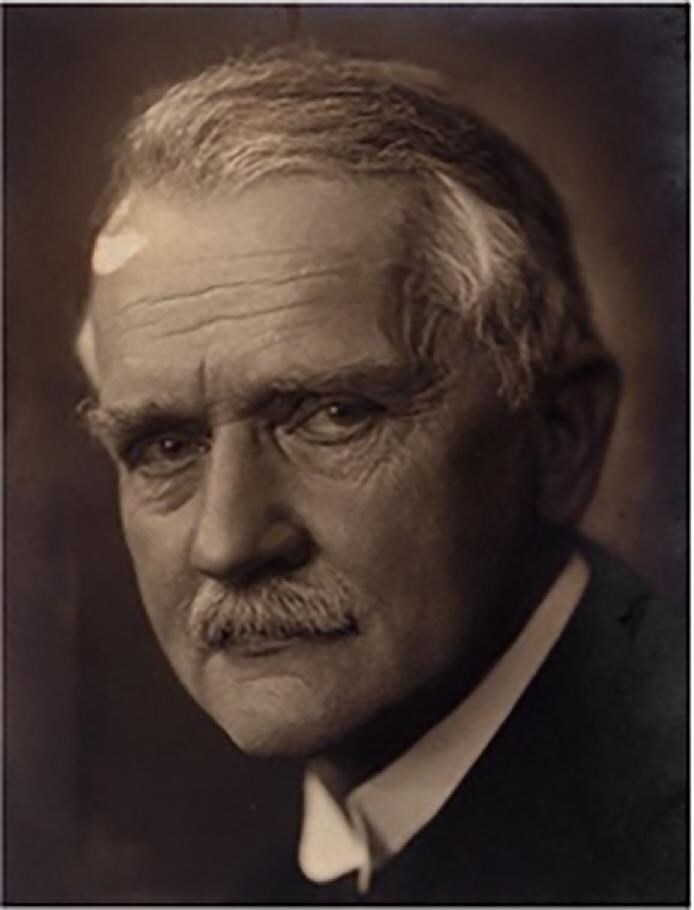
Wilhelm August Paul Schüffner. Source: Wellcome Collection 578821i (Public Domain Mark)^5^

**Figure 2.**
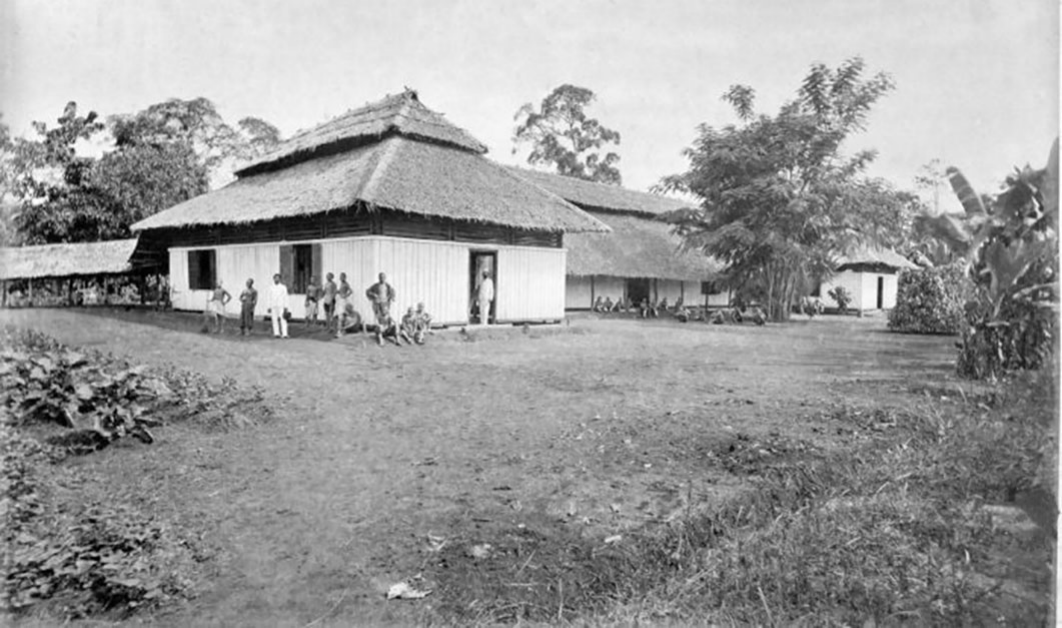
The Senembah Company Hospital at the tobacco plantation of Tanjung Morawa. Source: Tropenmuseum, CC BY-SA 3.0, via Wikimedia Commons^6^

**Figure 3.**
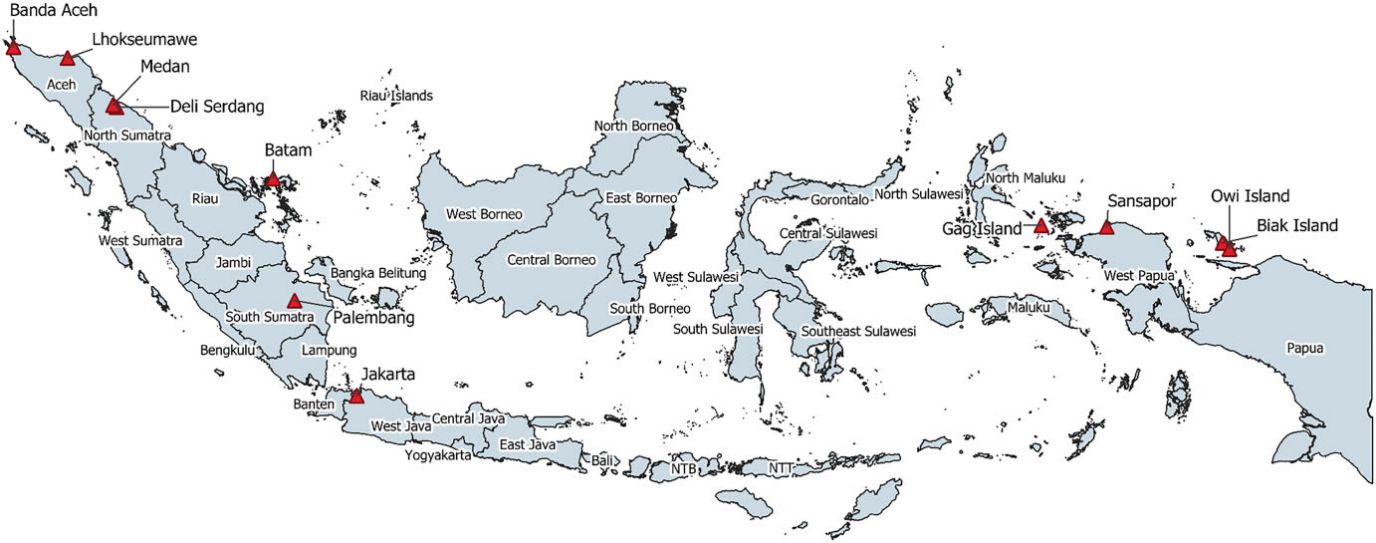
Map of Indonesia with provinces named and relevant locations referred to in the text annotated. The locations of interest are represented with red triangles. The base layer used to create this map was modified from the UN Food and Agriculture Organisation's Global Administrative Unit Layers.^9,10^ Deli Serdang is the current district name of the area that includes old Deli. NTB: Nusa Tenggara Barat (West Nusa Tenggara); NTT: Nusa Tenggara Timur (East Nusa Tenggara).

In 1902, Dr Wilhelm Schüffner (Figure 1, discoverer of ‘Schüffner's dots’ in *Plasmodium vivax* malaria) found cases similar to typhoid fever in the plantations of *Senembah Maatschappij* (Senembah Company), a tobacco company in Deli, Sumatra (Figures 2 and 3).^2,3^ Laboratory investigation showed that the aetiology differed from typhoid fever; however, due to the similarities, Schüffner referred to this disease as pseudotyphoid (*pseudotyphus* in Dutch).^2^ Severe cases progressed to the ‘typhus state’, with drowsiness, delirium and restlessness.^2^ Although pseudotyphoid of Deli had lower mortality, Schüffner also noted the similarities in clinical presentations and transmission with the Japanese Kedani fever, an entity now known to be scrub typhus.^2,3^ In 1908, Schüffner described an outbreak of 158 cases among 7700 workers in the plantations.^2,4^ In the Dutch East Indies, in addition to pseudotyphoid, ‘mite fever’ was also used to describe scrub typhus. In his 1927 publication, van Driel used the term *mijtekoorts* (mite fever).^11^

Based on patients’ histories, Schüffner suspected that exposure to ticks or mites might be involved, specifically the ‘Trombidium of Deli’ (later named taxonomically as *Leptotrombidium deliense*).^2^  *L. deliense*, as a vector associated with outbreaks, was first described by Walch in 1922.^7,8^

Scrub typhus was also reported among military personnel. In 1932, Emanuels^12^ reported cases in the military hospital in Banda Aceh, Sumatra, described as ‘tropical typhus’ and ‘mite fever’, defined as having a positive Weil-Felix OXK test and an eschar, respectively. These patients were soldiers and accompanying Indonesian labourers who had returned from patrol in the Seulimeum, Kroëng Raja (Krueng Raya) and Lho-nga (Lhoknga) area.^12^ Kotter^13^ also reported how from 1932 to 1937 more patients died from mite fever (four) than from malaria (one). Then, in January 1938, mite fever paralyzed a brigade with febrile illness for nearly 2 months.

**Figure 4.**
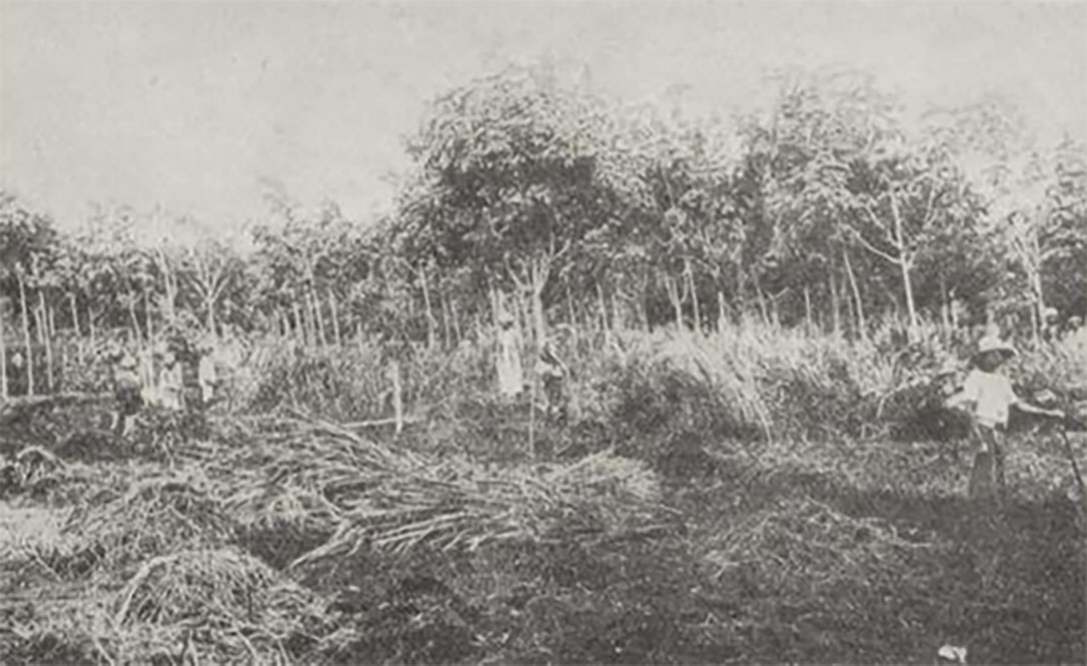
Workers clearing the overgrown area within the plantation. Source: Walch and Keukenschrijver, Geneeskundig Tijdschrift voor Nederlandsche Indie 1924, Vol. 64, Issue 2, page: 250–251^4^

In 1941, van der Schroeff, based in the military hospital in Lhokseumawe, Sumatra, also reported mite fever and rural typhus in palm oil plantation workers.^14^ These workers were tasked to clear an abandoned coffee plantation overgrown with *lalang* (tall grasses) (Figure 4).^14^ In the 1930s, scrub typhus was documented in other parts of Indonesia, including Bengkulu, West Borneo, Jakarta and Banten.

The earliest documentation of scrub typhus in Indonesia was mostly associated with the economic and military interests of the Dutch colonial government. As with other tropical diseases, the study and treatment of scrub typhus were fundamentally to safeguard colonial interests rather than the well-being of the native population.^15^ The Dutch colonial authorities recognised that understanding and mitigating tropical diseases held strategic importance, as it could enhance the productivity of the workforce, facilitate commodity extraction and reduce morbidity and mortality among military personnel. While the provision of some medical services to native communities did have the indirect effect of improving their health, this was a by-product. Scientific research, healthcare infrastructure and public health interventions were all designed to serve colonial prerogatives, focusing on the health needs of European colonisers rather than the native Indonesian populace. At times, colonial regimes justified their oppressive actions by citing a civilising mission to provide healthcare for the indigenous population. This paternalistic mindset reminds us of the origin of tropical medicine as a tool of colonial control.^16^

## World War II (1942–1945)

In 1942, the forces of Japanese Empire successfully invaded the Dutch East Indies, causing a power shift in Indonesia. The Japanese forces also invaded the island of New Guinea, which was under the administration of the Netherlands (western part) and Australia (eastern part)—now respectively the Indonesian Papua provinces and the country of Papua New Guinea.^17^

In 1945, Griffiths^20^ reported a scrub typhus outbreak among Allied forces in Dutch New Guinea. The first case was diagnosed on 6 August 1944. By 30 September 1944, there were 931 cases and 34 fatalities. The majority of cases (>500) caught scrub typhus in ‘semi-cleared areas’ that might have been abandoned villages or plantations. Other patients contracted scrub typhus in ‘overgrown coconut groves’ and the meeting of beach and forest. The soldiers also deployed several disease control efforts, including clearing the areas by removing grass and scrub, ‘rat control’ and treating clothing with insecticides. In 1947, Griffiths^21^ published a follow-up paper reporting on finding *L. deliense* in Sansapor.

Scrub typhus was the leading cause of deaths by communicable diseases among the American forces in Southeast Asia and therefore threatened military operations.^18^ Among the American, British and Australian troops, scrub typhus had caused >16 000 cases and >600 fatalities;^18^ in perspective, malaria had caused 113 256 cases and 90 deaths in the US Navy and Marine forces.^19^ Based on ‘fragmented’ recovered medical records and prisoners’ interviews, it is reasonable to believe that scrub typhus also affected the Japanese forces in ‘epidemic proportions’.^18^ The Japanese did not realise until late 1944 that they had encountered the same disease as tsutsugamushi disease (as they called what is now generally termed scrub typhus) in Japan.^18^

The medical research and advancements that took place during World War II were inextricably tied to the geopolitical objectives of the warring parties. Scientific research and military medicine were harnessed as strategic tools to strengthen fighting capabilities. ‘The urgency, aura of crisis, national attention, and material resources’ available to deploy in wars have ‘catalysed’ advancements in medicine, such as the development of penicillin.^22,23^ Although penicillin was not effective against scrub typhus, understanding tropical diseases, including scrub typhus, would provide the Allied forces a tactical advantage.

## The Indonesia national awakening and Old Order era (1945–1966)

Following the end of World War II, geopolitical factors led to a reduction of research on scrub typhus during the unstable period that followed Indonesia's declaration of independence on 17 August 1945. This was contested by the returning Dutch colonial authorities who began a military campaign to regain control of its former colonial territory. Finally, on 27 December 1949, the Dutch government accepted the sovereignty of most of Indonesia, keeping only Dutch New Guinea as a colonial possession until 1962.

In 1950, Plooij, from the military hospital in Palembang, South Sumatra, reported 40 scrub typhus cases after exposure to *alang-alang* (*lalang*) fields.^24^ Plooij tried to treat the patients with para-aminobenzoic acid (PABA), which was said to have some effect, however, it was not as significant compared with previous documentation in the literature.^24^ In the early 1950s, Hoogerheide and Ensink documented two clusters of cases on the islands Biak and Noessi (Nusi) off the north coast of Dutch New Guinea (Papua).^32^ Patients were treated with chloromycetin (chloramphenicol) and all recovered.^25^

Aside from several studies in Dutch New Guinea, there is a gap in the data on scrub typhus from 1951 to 1970. Indonesia faced many political and economic challenges in the early period of independence. In 1965, an attempted coup d’état was followed by anti-communist purges from late 1965 to early 1966, during which hundreds of thousands were killed. Ultimately, in 1967, Sukarno—the first president—was overthrown and replaced by General Suharto. In 1962, Indonesia started to govern western New Guinea under the supervision of the United Nations. During July and August 1969, a plebiscite involving 1025 indigenous people chosen by the Indonesian authorities voted unanimously for the integration of western New Guinea into Indonesia. Months later, western New Guinea was formally united into the Republic of Indonesia and named Irian Jaya province.

The global, regional and national upheavals of the mid-20th century almost completely disrupted the conduct of scientific research for an entire generation. They contributed substantially to the paucity of evidence on scrub typhus in Indonesia and low awareness of the disease among medical staff and health authorities. Those effects linger in contemporary Indonesia with specific regard to the extreme paucity of investigations on the distribution and burdens of scrub typhus.

## The New Order era (1967–1998)

Suharto's appointment as the second president in 1967 marked the start of the New Order era. His authoritarian regime brought Indonesia into a relatively stable period that was focused on socio-economic development that was accompanied by an increase in corruption and cronyism.

In the 1970s, Indonesia had become more stable and medical research was starting to resume. One of the institutions that worked on scrub typhus was the Centre for Disease Vector and Reservoir Research and Development (Balai Besar Penelitian dan Pengembangan Vektor dan Reservoir Penyakit [B2P2VRP]) under the Ministry of Health of the Republic of Indonesia.^26^ The B2P2VRP collected *Leptotrombidium* vector specimens from 1971 to 2011 in Sumatra (North Sumatra, Jambi, Bengkulu, Lampung), Java (Banten, Jakarta, West Java, Central Java, East Java), South Borneo and Papua, including *L. deliense, L. fletcheri, L. akamushi, L. arenicola, L. imphalum* and *L. scutellare.*^27,28^

**Figure 5.**
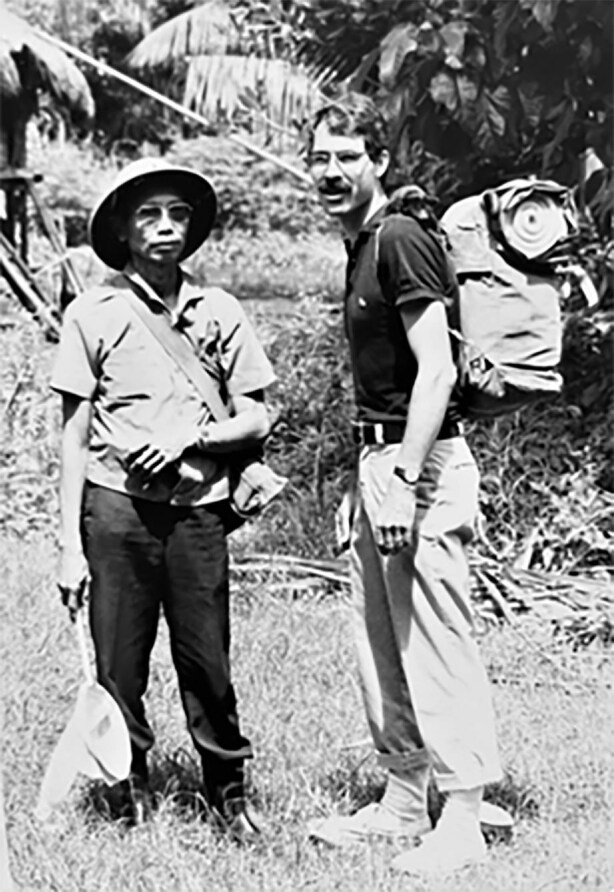
David Dennis and Soeroto Atmosoedjono (NAMRU-2 Jakarta) pictured during field investigation of scrub typhus vector. Photograph courtesy of David Dennis.

The US Naval Medical Research Unit No. 2 (NAMRU-2) undertook research on scrub typhus in Indonesia and elsewhere in the region. The Jakarta Detachment of NAMRU-2 was established in 1970 through a bilateral agreement between the US and Indonesian governments intended to last 30 y.^29^ NAMRU-2 operated in Indonesia for approximately 40 y and produced ‘substantial contributions to local, global, and military medicine’, including building capacity and laboratory facilities.^29^ NAMRU-2 also participated in public health interventions such as the malaria control effort in Central Java, the digital Early Warning Outbreak Recognition System (EWORS) and avian influenza (H5N1) diagnosis.^29^ NAMRU-2, in collaboration with the Indonesia National Institute of Health Research and Development, has contributed considerably to scrub typhus research in Indonesia. Among 51 articles with primary data on scrub typhus in Indonesia included in Saraswati et al.,^1^ 14 (26.9%) articles listed authors from NAMRU-2. Figure 5 showcases the Indonesia–US research cooperation. From 1975 to 1985, various studies on vectors and non-human hosts were conducted in East Java, Jakarta, Biak and Owi Island off the north coast of Papua, West Sumatra, West Java, Batam Island (about 10 km south of Singapore) and Central Sulawesi.^30–38^ The work of NAMRU-2 in Indonesia ended in 2010 due to a complex array of political and social issues (more explanation under the Reform era section).^39^

In addition to NAMRU-2, other foreign institutions including the Smithsonian Institution, Indiana State University, Vanderbilt University and University of Hawaii performed studies on scrub typhus—mainly on the vectors.^40–42^

**Figure 6.**
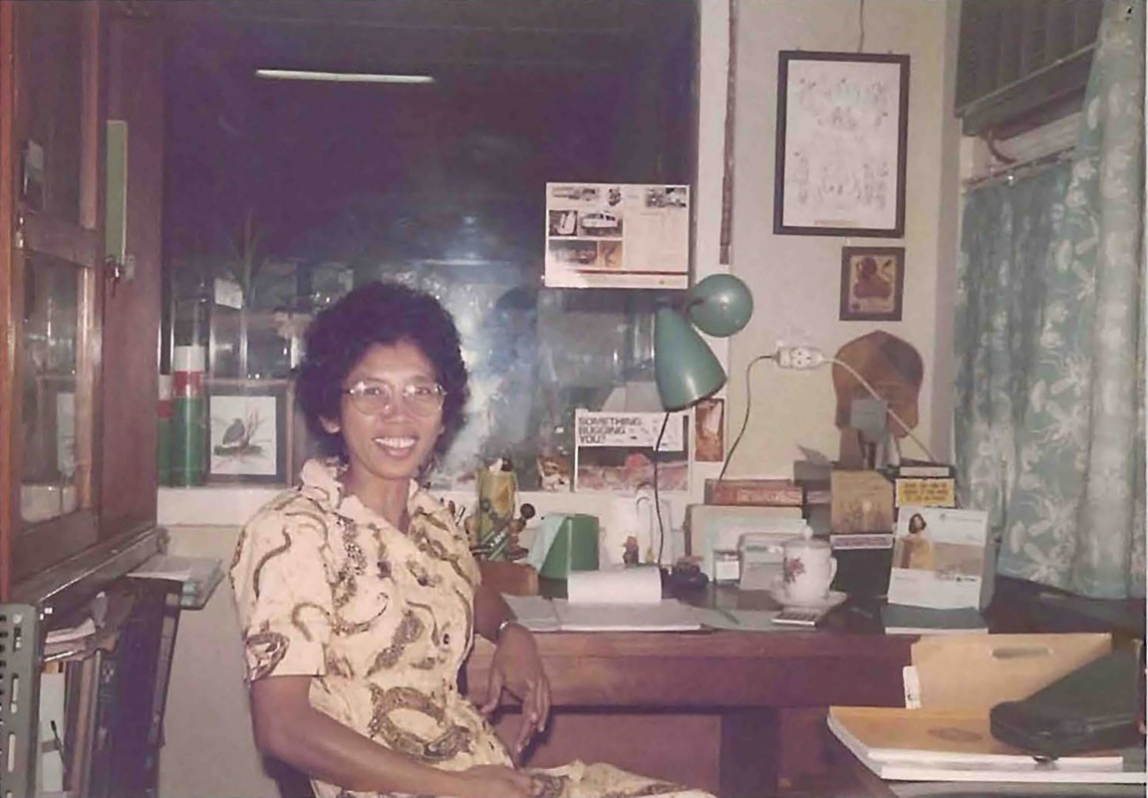
Tuti Hadi, pioneering Indonesian acarologist, pictured in her office. Photograph courtesy of Lenny Ekawati

During the 1970s, the Indonesian government expanded a so-called transmigration program. This involved the relocation and settlement of people from the more populated islands of Java, Bali and Lombok to the underdeveloped and sparsely populated outer islands in direct connection with recently opened agricultural sites.^43^ Various studies on the transmigrant communities, non-human hosts and vectors in South Sumatra, Jambi and Lampung were performed. Hadi (Figure 6), a pioneering Indonesian acarologist, contributed substantially to scrub typhus research in Indonesia.^30–38^,^44–47^

The military populations were also studied during this period. In a 1992–1993 survey, Corwin et al.^48^ investigated rickettsial infections in Indonesian military personnel deployed to Cambodia for United Nations peacekeeping duties and reported scrub typhus seropositivity of 7.9%. Richards et al.^49^ also surveyed military communities in Malang, East Java, in 1994 and found scrub typhus antibodies in 5.1% of trapped rodents and 1.3% of human participants.

Studies were also conducted in more urban areas. In 1975, Dennis et al.^50^ performed an immunofluorescence assay survey of a community at Ancol in northern Jakarta, finding seroprevalence of scrub typhus at just 0.36%.^31^ Richards et al.^49^ also reported one acute case of scrub typhus (immunoglobulin G [IgG] enzyme-linked immunosorbent assay positive) in Jakarta.

## The Reform era

After the fall of Suharto, Habibie assumed the presidency. During this period, there was a shift towards a more democratic society. Habibie conducted various political reforms, including passing the Regional Autonomy law that decentralised power.

Indonesia's transition into democracy, marked by the resignation of Suharto in 1998, set off a chain of events leading to the end of NAMRU-2 cooperation, as it was viewed as Suharto's legacy.^29^ In November 1998, Indonesia's Minister of Defense and Commander of the Armed Forces proposed to the Minister of Foreign Affairs to terminate cooperation with NAMRU-2.^51^ Several Indonesian officials also started to express their concerns that NAMRU-2 was conducting ‘intelligence activities’, although this was denied.^51^

In 2005, NAMRU-2 detected the first human case of H5N1 (avian influenza) in Indonesia.^51^ However, in 2007 the Ministry of Health ‘took over control’ of H5N1 diagnosis from NAMRU-2 and Indonesia stopped sharing clinical samples with a World Health Organization–affiliated laboratory network.^39,51^ This ‘drastic action’ was prompted by the unauthorised sharing of samples with a database at Los Alamos National Laboratory and pharmaceutical companies.^51^ The results of analyses performed on the shared Indonesian samples were presented without the knowledge of the Indonesian government.^39^ The Health Minister was also informed that an Australian company was developing a vaccine using the shared virus strain.^51^ Bilateral negotiations to keep NAMRU-2 operating were not fruitful and NAMRU-2 operations were finally halted in 2010.^51^

During this period, from 2007 to 2008, Widjaja et al.^52^ (NAMRU-2) studied the distribution of rickettsiosis in Java, Sumatra, Sulawesi and Borneo. In North Sulawesi and East Borneo, they detected *Orientia tsutsugamushi* in *Leptotrombidium* mites using polymerase chain reaction.^52^ They also found positive antibodies against *O. tsutsugamushi* in trapped small mammals (rodents) across North Sulawesi, West Sumatra and East Borneo.

Between 2013 and 2016, a study was carried out at eight tertiary hospitals in Java, Bali and Sulawesi to investigate acute febrile illness.^53^  *O. tsutsugamushi* IgG was positive (3.8% [n=19/504]) across Jakarta, West Java, Yogyakarta, Central Java, East Java and South Sulawesi. However, no cases of acute scrub typhus were observed.

## Future outlook

In its earliest days, the knowledge generation process of understanding scrub typhus started as an effort to assist colonial interests. However, Indonesia now has an opportunity to take ownership and focus its health research and knowledge production to better address the needs of its population, especially the ones in limited-resource settings most affected by scrub typhus. Investing in strengthening the country's overall health system, diagnostic capacity and surveillance and building up local scientific capacity should be prioritised. This can help equip Indonesia to control scrub typhus and eventually improve the health of its communities.

Recent policy developments have raised uncertainties regarding the future of scientific research in Indonesia. In 2021, in a move that shook up the entire science system, the Badan Riset dan Inovasi Nasional (National Research and Innovation Agency [BRIN]) was created as a ‘science super-agency’, absorbing research centres embedded in ministries and government agencies.^54,55^ This formation was intended to improve efficiency and coordination in research funding and administration.^55^ However, formation of the BRIN has been met with concerns of political overreach in scientific research.^55,56^ Three years on, researchers have voiced that the research and administrative process have become more ‘bureaucratic’.^56^

Indonesia's newly inaugurated president and his administration is committing an additional US$31 million for research.^57^ The new administration is also forming a new ministry, the Kementerian Pendidikan Tinggi, Sains, dan Teknologi (Ministry of Higher Education, Science, and Technology), which will also address the development of university research infrastructure. We hope that the increased budget and reorganisation of research agencies can help improve the amount and quality of research done in Indonesia and ultimately help improve our understanding of scrub typhus.

## Conclusions

Although scrub typhus in Indonesia has been studied since the early 1900s,^1^ there has been limited fundamental research and surveillance studies. The existing knowledge gaps, partly attributed to limited diagnostic capacity and insufficient awareness among healthcare professionals, present challenges for policymakers in grasping the burden and significance of scrub typhus. Comprehensive studies with robust sampling methods are necessary to generate data on exposure and cases through employing seroepidemiology and acute febrile illness studies. Establishing scrub typhus as a notifiable disease with a sustainable surveillance system and securing adequate funding are vital for understanding the transmission dynamics over time. The implementation of a national guideline on scrub typhus and recognising it as a neglected tropical disease would improve awareness and potentially open access to more funding for research and disease control. Once the burden of scrub typhus is acknowledged, integrating it in the list of differential diagnoses for empiric treatment of acute febrile illnesses can be facilitated.

Looking at the history of scrub typhus in Indonesia, we can appreciate how geopolitical upheavals disrupted scientific progress and created significant and lasting gaps in knowledge. Today, Indonesia is a comparatively stable and prosperous democratic nation endowed with a nascent and rapidly developing capacity for biomedical research. Those may be usefully directed to the poorly defined burdens of morbidity and mortality and to the ecological and geographic distributions of scrub typhus.

## Data Availability

The data underlying this article are available in the article.
